# Impact of Pre-Operative Ureteroscopy on Bladder Recurrence Following Nephroureterectomy for UTUC

**DOI:** 10.3390/cancers16152683

**Published:** 2024-07-28

**Authors:** Chris Ho-Ming Wong, Ivan Ching-Ho Ko, David Ka-Wai Leung, Kang Liu, Hongda Zhao, Mario Alvarez-Maestro, Maria del Pilar Laguna Pes, Jean de la Rosette, Jeremy Yuen-Chun Teoh

**Affiliations:** 1 S.H. Ho Urology Centre, Department of Surgery, Faculty of Medicine, The Chinese University of Hong Kong, Hong Kong, China; 2 Department of Urology, Hospital Universitario La Paz, 28046 Madrid, Spain; 3 Department of Urology, Medipol Mega University Hospital, Istanbul Medipol University, Istanbul 34083, Türkiye; 4 Department of Urology, Medical University of Vienna, 1090 Vienna, Austria; 5 Li Ka Shing Institute of Health Sciences, The Chinese University of Hong Kong, Hong Kong, China

**Keywords:** upper tract urothelial carcinoma, ureteroscopy, nephroureterectomy, survival outcomes, bladder recurrence

## Abstract

**Simple Summary:**

This research investigates whether a diagnostic technique called ureteroscopy (URS), performed before surgery for removing the kidney and ureter, influences the likelihood of cancer recurrence in the bladder in patients with upper tract urothelial carcinoma (UTUC). Data from a multicentre international registry were analysed to compare patients who underwent URS before their surgery with those who did not. The study found that patients who had URS prior to surgery experienced a higher rate of cancer recurrence in the bladder. These results highlight the need for careful consideration of the use of URS in the diagnostic process for UTUC, as it could affect long-term outcomes. This information is crucial for clinicians in optimizing treatment strategies and improving patient care.

**Abstract:**

(1) Introduction: Diagnostic ureteroscopy (URS) is an important component in the workup of upper tract urothelial carcinoma (UTUC). Whether URS was associated with increased recurrence in the bladder was not fully concluded. The current study aimed to evaluate the implication of URS on the incidences of intravesical recurrence following radical nephroureterectomy (RNU) in non-metastatic UTUC patients without prior history of bladder cancer via multi-institutional data. (2) Patients and Methods: Data were obtained from the Clinical Research Office of the Endourology Society Urothelial Carcinomas of the Upper Tract (CROES-UTUC) registry, a prospective, multicentre database. Patients with non-metastatic UTUC treated with RNU were divided into two groups: those undergoing upfront RNU and those having diagnostic URS prior to RNU. Intravesical recurrence-free survival (IVRS) was the primary endpoint, evaluated through Kaplan–Meier analysis and multivariate Cox regression. Cases with adequate follow-up data were included. (3) Results: The analysis included 269 patients. Of these, 137 (50.9%) received upfront RNU and 132 (49.1%) received pre-RNU URS. The URS group exhibited an inferior 24-month IVRS compared to the upfront RNU group (HR = 1.705, 95% CI = 1.082–2.688; *p* = 0.020). Multivariate analysis confirmed URS as the only significant predictor of IVR (*p* = 0.019). Ureteric access sheath usage, flexible ureteroscopy, ureteric biopsy, retrograde contrast studies, and the duration of URS did not significantly affect IVRS. (4) Conclusions: Diagnostic URS prior to RNU was found to be associated with an increased risk of IVR in patients with UTUC. The risk was not significantly influenced by auxiliary procedures during URS. Physicians were advised to meticulously evaluate the necessity of diagnostic URS.

## 1. Introduction

Upper tract urothelial carcinoma (UTUC) represents a relatively rare malignancy, accounting for approximately 5–10% of all urothelial cancers [1]. The management of UTUC poses distinct challenges, notably those associated with achieving an accurate diagnosis [2]. While ureteroscopy (URS) has been a key part of the diagnostic algorithm, a particular concern is the high rate of intravesical recurrence (IVR) that manifests after the curative treatment of radical nephroureterectomy (RNU). It substantially affects patient quality of life and necessitates further treatments [3].

Multiple international guidelines endorse diagnostic URS as an invaluable tool for assessing UTUC. It allows for direct visualization of the upper urinary tract and histopathological confirmation through biopsy [4]. However, these guidelines concurrently highlight the potential for tumour seeding during URS, which may elevate the risk of IVR post-RNU [5]. This scenario presents a clinical conundrum for urologists who must weigh the necessity of URS against the risk of increased bladder recurrence.

The influence of diagnostic URS on IVR following radical RNU remains a subject of debate, with studies yielding disparate findings [6,7]. Although existing systematic reviews have described the relationship between URS prior to radical surgery and bladder recurrences, the included studies are predominantly single-centre and exhibit considerable variability [5,6]. Additionally, many of these investigations lack comprehensive details regarding the ureteroscopic techniques utilized, which hampers their ability to definitively ascertain the impact of URS on IVR.

The current study seeks to fill in this knowledge gap via utilising data from a multicentre, international study—the Clinical Research Office of the Endourology Society Urothelial Carcinomas of the Upper Tract (CROES-UTUC) registry. The authors aim to explore the association between pre-operative diagnostic URS and intravesical recurrence-free survival (IVRS) among patients undergoing RNU for UTUC.

## 2. Patients and Methods

### 2.1. The CROES-UTUC Registry

Data for the present analysis were sourced from The Clinical Research Office of the Endourology Society Urothelial Carcinomas of the Upper Tract (CROES-UTUC) registry. Established in 2014, this registry is one of the largest real-world, prospective, global databases in UTUC management, incorporating contributions from 29 participating centres across 101 countries. It is registered on ClinicalTrials.gov (NCT02281188) [8] and adheres to the study protocol published according to the Agency for Healthcare Research and Quality guidelines for the design and use of patient registries for scientific, clinical, and health policy purposes [9,10].

This study included consecutive patients over the age of 18 diagnosed with non-metastatic UTUC and treated with radical nephroureterectomy. Only cases with sufficient follow-up data and results of cystoscopic examinations were included. Exclusion criteria included: concomitant or history of bladder cancer, a history of nephron-sparing surgery on the ipsilateral side as the RNU, therapeutic interventions performed during the same session as the ureteroscopic examination, or history of neoadjuvant therapy. Diagnostic workups, operative procedures, and follow-up protocols were not standardised, but were provided according to the standard of care at each participating centre.

### 2.2. Data Collection and Analysis

Patient baseline characteristics, disease details, treatment information, and follow-up data were documented. For those undergoing diagnostic ureteroscopy, details such as the use of retrograde contrast studies and post-operative ureteric stent placement were analysed. Follow-up procedures, including check cystoscopies conducted up to 24 months post-operation, were thoroughly recorded and analysed. The reported tumour grading adhered to the World Health Organization classifications of 2004 and 2016. Disease staging was based on pathological examination of the RNU specimen. Data collection was facilitated by the online Data Management System, a web-based platform located at the CROES Office.

Patients were categorised into two groups: (1) those undergoing upfront radical nephroureterectomy and (2) those undergoing diagnostic ureteroscopy prior to surgery. The primary endpoint analysed was intravesical recurrence-free survival (IVRS), with vesical recurrence determined by check cystoscopy with histological confirmation. Events of extra-urothelial recurrence following RNU that did not involve vesical recurrence were excluded from the endpoint analysis.

### 2.3. Statistical Methodology

Statistical analyses were conducted using SPSS version 25.0 (IBM, New York, NY, USA). Differences in categorical variables between groups were assessed using Pearson’s chi-square test or Fisher’s exact test as appropriate, while continuous variables were evaluated using the Mann–Whitney U test in a non-parametric fashion. Kaplan–Meier analysis with log-rank test was adopted to assess IVRS. Univariate and multivariate Cox regression analyses (proportional hazards regression) were performed to identify potential confounders influencing IVRS. Variables included in the multivariate analysis were those identified as either significant (*p* < 0.05) or near-significant (*p* < 0.2) in the univariate analysis, known contributory factors documented in existing literature, or those that existed as baseline discrepancies between groups. All statistical tests were two-sided, with a *p*-value of less than 0.05 considered statistically significant.

## 3. Results

Upon the application of the inclusion and exclusion criteria, a cohort of 269 cases with adequate follow-up data was analysed. Of these, 137 cases (50.9%) belonged to the upfront radical nephroureterectomy (RNU) group, while the remaining 132 patients (49.1%) were categorised into the ureteroscopy group. The median follow up duration of the upfront RNU group was 16.8 months, while that of the ureteroscopy group was 15.7 months. Both groups exhibited similar demographic and clinical characteristics. The median age in the upfront RNU group was 62.3 years compared to 66.4 years in the ureteroscopy group; however, this age difference was not statistically significant (*p* = 0.188). Preoperative hydronephrosis was more prevalent in the ureteroscopy group (37.1%) than in the upfront RNU group (25.7%). Other factors, including multifocality, high-grade tumours, approach of RNU, use of open bladder cuff excision, and use of adjuvant chemotherapy post-RNU, were comparable between groups.

Within the ureteroscopy group, a biopsy of the lesion was performed in 54.5% of patients. A retrograde contrast study was conducted in 61.4% of cases, and a ureteric access sheath was used in 6.8% of the group. Following diagnostic ureteroscopy, 40.9% of patients received a ureteric stent while awaiting their definitive operation. Comprehensive details of these interventions are summarised in Table 1.

**Figure 1:**
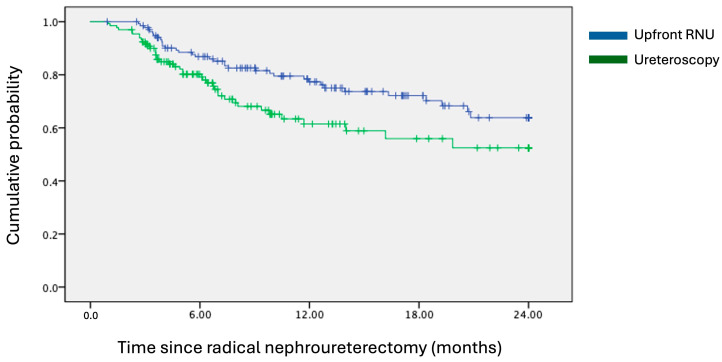
Kaplan–Meier survival curves of 24-month intravesical recurrence-free survival. Hazard ratio = 1.705; 95% CI = 1.082–2.688; *p*-value = 0.020.

Kaplan–Meier survival analysis revealed that the ureteroscopy group was associated with inferior 24-month IVRS, with a hazard ratio of 1.705 (95% CI = 1.082–2.688; *p* = 0.020), as depicted in Figure 1. At 24 months, the IVRS rates were 64% for the upfront RNU group and 52% for the ureteroscopy group. In the univariate analysis of potential confounding factors, diagnostic ureteroscopy was the only variable found to significantly predict a poorer primary outcome (*p* = 0.022). Other factors, such as open bladder cuff excision (*p* = 0.119), multifocal ureteric tumours (*p* = 0.137), and post-RNU bladder instillation of chemotherapy (*p* = 0.064), were included in the multivariate analysis model as they were of near statistical significance (*p* < 0.2). Pre-operative hydronephrosis was also analysed due to discrepancies between the two groups. In the multivariate analysis, ureteroscopy consistently emerged as the sole statistically significant predictor of increased risk of intravesical bladder recurrence (*p* = 0.019). These findings are detailed further in Table 2. Additionally, various operative parameters during ureteroscopy were evaluated; however, the deployment of a ureteric access sheath (*p* = 0.582), use of flexible ureteroscopes (*p* = 0.876), performance of a biopsy on the ureteric lesion (*p* = 0.366), undertaking of a retrograde contrast study (*p* = 0.964), and the duration of ureteroscopy (*p* = 0.593) did not significantly impact IVRS.

## 4. Discussion

The implications of performing ureteroscopy prior to radical nephroureterectomy have not been thoroughly investigated. In our multicentre study, we identified the use of ureteroscopy (URS) as an independent predictor of intravesical recurrence post-nephroureterectomy. Overall, our results echo the current European Association of Urology (EAU) guidelines and are supported by meta-analyses from 2017 and 2018, which reported pooled hazard ratios of 1.51 and 1.56, respectively, underscoring an increased risk of bladder recurrence following diagnostic ureteroscopy [5,6] in patients due for curative RNU.

The survival curve analysis suggests a two-stage recurrence pattern: a significant portion of early recurrence develops within the first year post-RNU (suggested by an initial steeper decline of the curve), followed by delayed recurrence in remaining cases. This pattern aligns with existing theories on intravesical recurrence (IVR) following RNU for upper tract urothelial carcinoma (UTUC), where both monoclonal and mixed clonal diseases have been observed in multifocal urothelial carcinoma [11]. One hypothesised mechanism involves downstream urothelial seeding from descending tumour cells [12], typically resulting in early-to-mid-term recurrence. Another theory described pan-urothelial carcinomatous changes, suggesting that bladder recurrence from this mechanism would be independent of UTUC interventions and, thus, less likely to occur early following RNU.

The issue of post-URS bladder recurrence is complex. This is somewhat exacerbated by the degree of variability that a procedure of diagnostic URS entails. In our analysis, we found that auxiliary procedures such as ureteric access sheath deployment, flexible ureteroscopy, ureteric biopsy, and retrograde contrast study do not contribute to bladder recurrence. In this regard, the more recent studies present conflicting results. A single-centre retrospective study of 834 RNU patients published in 2021 indicated that ureteroscopy without biopsy did not increase bladder recurrence risk [13]. In this study, Sharma et al. compared 442 patients receiving ureteroscopic biopsy with 210 patients receiving upfront RNU and 125 patients receiving ureteroscope without biopsy. They reported a hazard ratio of 1.40 for the ureteroscopic biopsy group in terms of bladder recurrence. Conversely, Chen et al. reported that ureteric biopsy is associated with a higher risk of bladder recurrence compared to non-biopsy ureteroscopy (*p* = 0.034) [14]. In our study, a total of 75 patients from the URS group underwent ureteric biopsy. There was no significant difference observed in bladder recurrence rates. Also, the role of increased manipulation and irrigation during ureteroscopy was hypothesised to increase tumour seeding and subsequent bladder recurrence [15,16,17]. However, our study did not find significant associations between the use of ureteric stents, retrograde contrast studies, and the duration of ureteroscopy with IVR outcomes. These contradictory findings highlight the variability in single-centre practices and underscore the importance of multicentre data in providing more generalisable outcomes. They also underscore the need for future prospective studies to clarify these relationships.

Moreover, earlier studies have shown mixed results regarding the impact of tumour characteristics on IVR. A systematic review and meta-analysis in 2015 linked higher tumour T stages to increased rates of IVR [18], while another meta-analysis found no correlation between tumour characteristics and bladder recurrence [19]. Our study did not identify tumour grading or staging as significant predictors of IVRS, suggesting that the relationship between tumour aggressiveness and vesical recurrence may be more complex and influenced by various factors.

Recently, there has been a reignition of interest in kidney-sparing surgery, especially endourological ablative therapies. The increasing popularity of the thulium fibre laser has been one of the propellors. Its wavelength of 1940 nm gives it the highest water absorption coefficient among all available medical lasers, and, hence, it is an excellent tool for soft tissue ablation [20,21]. Its prolonged pulse duration enables it to induce continuous and reasonable carbonisation to maintain haemostasis [22]. There are even reports of en-bloc resection of UTUC performed with thulium fibre and Thulium–YAG lasers [23]. The role of ureteroscopy in the management of UTUC can only become more important in the future. With technological advancements, endourologists will only be encouraged to test beyond the limits and perform more ureteroscopic procedures for UTUC. Against this backdrop, the issue of post-URS bladder recurrence will still be one of the concerns. Apart from URS adjuncts, the role of post-URS intravesical chemotherapy needs to be studied [24,25,26]. To date, such studies have focused only on local complications like ureteric recurrences and ureteric strictures. Our question regarding post-URS IVR remains not fully answered. More investigations will be needed to identify the mechanisms of this phenomenon.

Overall, the issue of whether ureteroscopy should be performed remains a complicated one. Features such as aggressive tumours on cross-sectional imaging, obvious obstructive effects with hydronephrosis, and positive self-void cytology should discourage the use of URS owing to the obvious limited benefits [27]. On the opposite spectrum, patients with equivocal imaging finding or those that may be candidates for same-session therapeutic URS with endoscopic treatment would be the ones that benefit from a URS. Meanwhile, those that fall in between would still require a thorough discussion between the attending physician and the patient before URS is performed [28]. Novel techniques such as photodynamic diagnosis and use of 5-aminolevulinic acid are some of the examples that may increase the yield of a diagnostic ureteroscopy should it be performed [29,30].

The strengths of this study include its multicentre and international design, which helps to mitigate the impact of practice variances across different centres. Data were meticulously collected using standardised electronic forms, providing detailed insights into tumour location, multifocality, and specifics of ureteroscopic procedures. However, the retrospective nature of the study and the similarity of the baseline characteristics between groups, while robust, do not completely rule out the influence of unobserved confounders. Another limitation of the current study is the lack of standardisation of how diagnostic URS was performed, owing to the numbers of centres involved in cohort formation. Therefore, controlling for the discrepancy between diagnostic URS procedures and adjusting the potential difference during analysis were not possible. Furthermore, the limited adoption of intravesical chemotherapy following RNU and the lack of data on operative details such as the rate early control of the ureter during RNU are other limitations of the current dataset.

Future prospective studies, ideally with multi-arm designs, addressing the nuances of URS and its auxiliary procedures are essential to further elucidate these findings and refine clinical practices.

## 5. Conclusions

This multicentre analysis demonstrated that diagnostic ureteroscopy prior to radical nephroureterectomy is associated with an increased risk of bladder tumour recurrence. Given these findings, physicians are advised to judiciously assess the necessity of performing diagnostic ureteroscopy in patients with UTUC. Additional benefits of preoperative ureteroscopy need to be anticipated in order to outweigh the potential drawbacks before one offers pre-operative URS prior to RNU.

## Figures and Tables

**Table 1:** Patient and disease characteristics.

	Upfront RNU		Ureteroscopy		*p* Value
	N	%/IQR/SD	N	%/IQR/SD	
Number of patients, %	137		132		
Median follow up duration (months), IQR	16.8	10.3	15.7	9.8	0.061
Median age, IQR	62.3	12.6	66.4	13.9	0.188
Gender, %					
Male	81	59.1%	94	71.2%	0.055
Female	55	40.1%	39	29.5%	
Mean BMI (m^2^/kg), SD	25.5	4.2	26.3	4.7	0.156
Patients with CKD, %	44	32.1%	54	40.9%	0.135
History of smoking, %	73	53.3%	77	58.3%	0.406
Pre-operative hydronephrosis during diagnostic URS, %	35	25.7%	49	37.1%	0.041
Use of flexible ureteroscope during diagnostic URS, %	N/A		54	40.9%	N/A
Use of access sheath during diagnostic URS, %	N/A		9	6.8%	N/A
Biopsy of lesion during diagnostic URS, %	N/A		75	56.8%	N/A
Retrograde contrast study during diagnostic URS, %	N/A		81	61.4%	N/A
Use of guidewire in in diagnostic URS, %	N/A		109	82.6%	N/A
Ureteric stent prior diagnostic URS, %	N/A		11	8.3%	N/A
Ureteric stent after diagnostic URS, %	N/A		54	40.9%	N/A
Tumour laterality, %					0.422
Left	69	50.4%	60	54.5%	
Right	68	49.6%	72	45.5%	
Multifocal tumour, %	15	10.9%	15	9.8%	0.74
Histology, %					0.302
Grade 1	11	8.0%	7	5.3%	
Grade 2	34	24.8%	31	23.5%	
Grade 3	84	61.3%	89	67.4%	
Missing	8	5.8%	5	3.8%	
pT stage, %					0.992
Ta/is	1	0.7%	3	2.3%	
1	48	35.0%	43	32.6%	
2	36	26.3%	36	27.3%	
3	50	36.5%	47	35.6%	
4	2	1.5%	3	2.3%	
Surgical approach of RNU, %					0.89
Open	43	31.4%	41	31.1%	
Laparoscopic or robotic	94	68.6%	91	68.9%	
Open bladder cuff excision, %	91	66.4%	87	65.9%	0.929
Post-RNU bladder instillation, %	15	10.9%	22	16.7%	0.182
Adjuvant chemotherapy, %	9	6.6%	13	9.8%	0.319

RNU = radical nephroureterectomy; SD = standard deviation; BMI = body mass index, URS = ureteroscopy; pT stage = pathology tumour staging; N/A = not applicable.

**Table 2:** Cox regression analysis of factors associated with intravesical recurrence-free survival.

Univariate Analysis	Effect Size	95% CI		*p* Value
Pre-operative ureteroscopy	1.705	1.082	2.688	0.022
Ureteric access sheath	0.642	0.155	2.66	0.541
Ureteric biopsy	0.823	0.439	1.541	0.542
Ureteric stent prior to ureteroscopy	0.728	0.179	2.966	0.658
Use of safety guidewire	0.737	0.349	1.554	0.423
Use of flexible ureteroscope	0.907	0.487	1.689	0.758
Duration of ureteroscopy	1	1	1	0.593
Retrograde contrast study during ureteroscopy	0.964	0.503	1.847	0.913
Ureteric stent after ureteroscopy	0.667	0.355	1.255	0.209
Hydronephrosis at diagnosis	0.979	0.596	1.607	0.932
pT stage				
Ta/Tis/T1	Ref			
T2	0.942	0.508	1.747	0.851
T3	1.501	0.881	2.558	0.135
T4	1.478	0.348	6.282	0.597
Tumour grade				
Grade 1	Ref			
Grade 2	0.897	0.25	3.216	0.867
Grade 3	2.036	0.638	6.505	0.23
Multifocal tumour	0.53	0.23	1.223	0.137
Adjuvant chemotherapy post-RNU	1.231	0.565	2.68	0.601
Bladder instillation post-RNU	0.386	0.141	1.056	0.064
Open bladder cuff excision in RNU	1.493	0.902	2.47	0.119
MIS for RNU	1.023	0.626	1.672	0.928
Smoking history	1.114	0.707	1.753	0.642
Age >70	1.205	0.765	1.898	0.421
Female	0.833	0.523	1.325	0.44
Existing renal impairment	1.27	0.803	2.008	0.306
**Multivariate Analysis**	**Effect Size**	**95% CI**		***p* Value**
Pre-operative ureteroscopy	1.761	1.099	2.82	0.019
Open bladder cuff excision	1.371	0.817	2.301	0.233
Multifocal tumour	0.955	0.457	1.995	0.902
Post RNU bladder instillation	0.39	0.141	1.078	0.07
Hydronephrosis at diagnosis	0.851	0.5	1.447	0.551

CI = confidence interval; pT stage = pathology tumour staging; RNU = radical nephroureterectomy.

## Data Availability

Data are available upon request submitted to the corresponding author.
